# Healthy Learning Mind - a school-based mindfulness and relaxation program: a study protocol for a cluster randomized controlled trial

**DOI:** 10.1186/s40359-016-0142-3

**Published:** 2016-07-11

**Authors:** Salla-maarit Volanen, Maarit Lassander, Nelli Hankonen, Päivi Santalahti, Mirka Hintsanen, Nina Simonsen, Anu Raevuori, Sari Mullola, Tero Vahlberg, Anna But, Sakari Suominen

**Affiliations:** Folkhälsan Research Center, Topeliuksenkatu 20, 00250 Helsinki, Finland; Department of Public Health, University of Helsinki, Helsinki, Finland; Institute of Behavioural Sciences, University of Helsinki, Helsinki, Finland; School of Social Sciences and Humanities, University of Tampere, Tampere, Finland; National Institute for Health and Welfare, Helsinki, Finland; Unit of Psychology, University of Oulu, Oulu, Finland; Department of Adolescent Psychiatry, Helsinki University Central Hospital, Helsinki, Finland; Department of Mental Health and Substance Abuse Services, National Institute for Health and Welfare, Helsinki, Finland; Department of Teacher Education, University of Helsinki, Helsinki, Finland; Department of Biostatistics, University of Turku, Turku, Finland; Department of Public Health, University of Skövde, Skövde, Sweden; Department of Public Health, University of Turku, Turku, Finland

**Keywords:** Children and adolescents, School-based intervention, Mindfulness, Health promotion, Mental health, Well-being

## Abstract

**Background:**

Mindfulness has shown positive effects on mental health, mental capacity and well-being among adult population. Among children and adolescents, previous research on the effectiveness of mindfulness interventions on health and well-being has shown promising results, but studies with methodologically sound designs have been called for. Few intervention studies in this population have compared the effectiveness of mindfulness programs to alternative intervention programs with adequate sample sizes.

**Methods/design:**

Our primary aim is to explore the effectiveness of a school-based mindfulness intervention program compared to a standard relaxation program among a non-clinical children and adolescent sample, and a non-treatment control group in school context. In this study, we systematically examine the effects of mindfulness intervention on mental well-being (primary outcomes being resilience; existence/absence of depressive symptoms; experienced psychological strengths and difficulties), cognitive functions, psychophysiological responses, academic achievements, and motivational determinants of practicing mindfulness.

The design is a cluster randomized controlled trial with three arms (mindfulness intervention group, active control group, non-treatment group) and the sample includes 59 Finnish schools and approx. 3 000 students aged 12–15 years.

Intervention consists of nine mindfulness based lessons, 45 mins per week, for 9 weeks, the dose being identical in active control group receiving standard relaxation program called Relax. The programs are delivered by 14 educated facilitators.

Students, their teachers and parents will fill-in the research questionnaires before and after the intervention, and they will all be followed up 6 months after baseline. Additionally, students will be followed 12 months after baseline. For longer follow-up, consent to linking the data to the main health registers has been asked from students and their parents.

**Discussion:**

The present study examines systematically the effectiveness of a school-based mindfulness program compared to a standard relaxation program, and a non-treatment control group. A strength of the current study lies in its methodologically rigorous, randomized controlled study design, which allows novel evidence on the effectiveness of mindfulness over and above a standard relaxation program.

**Trial registration:**

ISRCTN18642659. Retrospectively registered 13 October 2015.

## Background

In the contemporary society, children and adolescents have to deal with several stressors on daily basis. Stressors may arise from family-system disturbances, peer conflicts, school context, socio-cultural challenges, vulnerabilities to physical and mental health problems, or from living in the fast-paced, media-saturated and multi-tasking world that sets high demands for performance, success and competition [1]. Research suggests that sustained stress in childhood and adolescence has negative influence on mental health, general functioning, and specific learning-related factors, such as executive function and working memory [2]. Approximately one fourth of youth suffers from at least one mental disorder during the past year, and respectively, about one third suffers from any lifetime mental disorder. Anxiety disorders are the most frequent mental disorders in children and adolescents, followed by behavior disorders, the mood disorders and substance use disorders [3]. In Finland, approximately 14 % of children aged eight to nine years suffer from some kind of mental health problems, and this share steeply arises along with the onset of puberty to 15–25 % in adolescent population [4]. Psychiatric disorders are the most important disorder group that impairs adolescents’ functional ability [5], and perceived stress is shown to increase the risk of subsequent mental disorders and their symptoms [6–8]. Thus, there is a need for effective, disseminable strategies to protect children and youth from dysfunctional effects of stress.

During the last few years, research on mindfulness has increased, and extended from initially focusing only on adults to including children and adolescents as well. However, studies with methodologically sound designs are still lacking. To be able to indicate the significant beneficial effects of mindfulness practice also on children’s and adolescents’ health and well-being, research needs to shift toward large, well-designed studies with robust methodologies, and adopt standardized formats of interventions, allowing for replication and comparison of studies, to develop a firm evidence base [9].

## Mindfulness and health

Mindfulness refers to a non-condemning state of awareness and readiness to pay attention to the stream of experiences in the present moment [10]. The concept is rooted in Eastern contemplative traditions and was later developed as part of therapeutic applications in psychology and medicine, such as mindfulness-based stress reduction (MBSR) [10, 11], mindfulness-based cognitive therapy [12], dialectic behavior therapy [13], and acceptance and commitment therapy (ACT) [14, 15]. The beneficial elements of mindfulness are suggested to include e.g. attention regulation, body awareness, emotion regulation, and change in perspectives on the self and learning [16]. Research among adults has shown that mindfulness practices reduce negative states of mind, such as stress [17], and symptoms of anxiety and depression [18–20], as well as alleviate various medical conditions, such as chronic pain [10] , type 2 diabetes [21, 22] and attention-deficit hyperactivity disorder [23, 24].

Furthermore, research among adults has shown promising positive associations between mindfulness practice and health behaviours, such as smoking cessation [25, 26], decreased binge eating [27], and decreased alcohol and substance use [28]. Finally, practicing mindfulness has also been shown to produce positive effects on psychological well-being in healthy participants [29–31].

Recently, also brain imaging has been utilized to study the neural level effects related to mindfulness based practices or meditation. Changes are reported both in structural properties [32, 33] and in brain functioning [19, 34], especially related to attentional control [35] and emotion regulation [36, 37].

In the previous decade, interest started to spread to mindfulness based approaches with children and adolescents and international research has shown promising preliminary results both in clinical context [23, 38–42] and in non-clinical, school context [43–49].

## Mindfulness among children and adolescents in school setting

It has been reported that mindfulness interventions are acceptable for children and adolescents, as well as feasible, and that they improve for example attention, emotional reactivity and some areas of meta-cognition [1]. Mindfulness-based programs have improved school-aged children’s attention and teacher-rated social skills [45]. A school-based (RCT) study showed significant improvements in post-treatment measures of self-rated test anxiety, teacher rated attention, social skills, objective measures of selective (visual) attention but no sustained attention, as well as improved behavioral regulation, metacognition, and overall global executive control among children who started out with poor executive functions [43]. Correspondingly, in another study [44] adolescents with lower pre-intervention self-regulation were observed to experience greatest improvements in behavioral regulation, meta-cognition and executive function. Preliminary research has shown that school-based mindfulness intervention programs may also result in beneficial outcomes regarding the interaction and pedagogical atmosphere among both students and students and their teachers [50].

In the school setting, mindfulness interventions reach the whole age group, and through the equal reach may even act as a counterforce for the prominent development of increasing inequality between different groups (based on e.g. gender, learning difficulties, health challenges, or socioeconomic background), yet empirical evidence is lacking.

While cost-effectiveness and ease of implementation of mindfulness programs in schools are notable advantages, sufficient evidence is still lacking on the role of mindfulness in fostering resilience, mental health and well-being among children and adolescents, over and above existing approaches such as relaxation. The previous studies conducted among youth are still few in number [1, 9, 23, 38–40, 43–45, 51, 52], and their methodological shortcomings (e.g. small sample sizes without control groups and/or unstandardized mindfulness intervention programs) prevent making generalizations of the efficacy of these interventions [9]. For instance, it is not well understood whether the observed changes persist or what the short and long-term effects of mindfulness intervention are [40]. Further, the role of mindfulness in improving health behavior among adolescents is not well known [53, 54].

It might be at place to state here also that the Finnish school system offers exceptionally good possibilities for examining between-individual variation as the school-related variance is minimized due to the homogenous schools system of our country: All schools follow the national curriculum, private schools are almost non-existent, and majority of students go to the nearest school in their residential area. Also areal segregation is still rather low compared to other countries. Furthermore, all teachers receive university education which reduces the teacher-related variance.

## The aim of the study

The comprehensive aim of this ongoing trial is to examine the effects of mindfulness practices in strengthening children’s and adolescents’ internal resources that promote mental wellbeing, cognitive functions, psycho-physiological responses, academic achievement, health behavior, motivational determinants of practice compared to a standard relaxation program and a non-treatment group (waiting-list). The primary aim is to determine the effectiveness of the school-based mindfulness program on three main outcomes: resilience (RS14), existence or absence of depressive symptoms (RBDI), and experienced psychological strengths and difficulties (SDQ). Secondary outcomes include mindfulness, happiness, satisfaction with life, quality of life, positive and negative affects, compassion/self-kindness, the rumination, and stress. Other explored factors among children and adolescents are cognitive functions, psycho-physiological responses, academic achievement, health behavior, motivational determinants of practicing mindfulness, and class room social environment. The study will also explore equity of distribution of the primary outcomes in terms of social background, gender, and learning difficulties of the students. The results of the study will be presented according to the 2010 CONSORT statement [55] and its extension to cluster randomized controlled trials [56].

## Methods

### Trial design

The study is an ongoing cluster randomized controlled trial (RCT) with three arms. Eligible schools were randomly allocated either to an intervention, control or non-treatment groups. Clusters were school classes (grades 6, 7 and 8) and age gap was from 12 to 15 years olds. The data collection started in the spring 2014, and finishes in the autumn 2016. The analyzing and reporting of the data starts in the autumn 2016.

### Randomization procedure

The recruitment started by listing all the schools in a Southern part of Finland. After choosing the schools (including as many classes of the same grade as possible), a letter explaining the study procedure was sent by e-mail to the head masters. Within few days after sending the information letter, the research team members called the headmasters by telephone. In most schools the decision to take part to the study was made collectively by the head master and the class teachers (of the chosen grades). The schools were enrolled from 14 cities/municipalities during the collection of the data (years 2014–2016). Altogether 247 schools were contacted, 59 of those participated in the study participation percentage being 24. In each municipality we aimed at an equal number of intervention and control classes. In order to achieve balanced intervention and control groups, schools participating in the study were randomized using the available background variables. The selection of intervention-control pairs was primarily based on the language being used for teaching (Finnish, Swedish or English, the grade, the school location, the number of classes participating in the investigation and, if necessary, the average apartment price per square meter in the school’s neighborhood).

The classes were randomly assigned to mindfulness intervention classes (*N* = 85) and control classes (*N* = 79) and non-treatment classes (*N* = 28). Due to practical reasons, in spring 2014 and in autumn 2015 schools were divided into two arms (intervention and control) and in spring 2015 and spring 2016 into three arms instead of two: intervention, control and non-treatment groups. First, the schools were divided into three groups based on the school location and the average apartment price per square meter. Within each of these groups, the total number of schools and classes varied. Next, the schools for these groups were divided into three subgroups including approximately same number of classes (some schools were combined into one subgroup to achieve an as even distribution of classes as possible).

### Data collection timeline

The data from intervention and control groups have been collected during four academic terms: In the beginning of spring term in 2014 (*N* = 523), in the beginning of autumn term in 2014 (*N* = 1090), in the beginning of spring term in 2015 (*N* = 821), and in the beginning of spring term in 2016 (on going, baseline including *N* = 203). Hence the last follow-up will be collected in spring 2017 (12 months follow-up of the spring 2016). Among intervention and control groups data have been collected at baseline, in the middle of the intervention (the fifth week of the intervention, a short formula), within 1 week after the intervention, and 6 and 12 months after baseline from the same participants.

Due to practical reasons, the data from non-treatment group have been collected during two academic terms: In the beginning of spring term in 2015 (*N* = 254), and in the beginning of spring term 2016 (ongoing, baseline including *N* = 109). Additionally, non-treatment group did not fill in the short formula in the middle of the intervention the measurement points being otherwise identical with the other two groups (incl. follow-ups).

Among teachers and parents data have been collected at baseline, after the intervention and 6 months after the baseline from the same parent (if only one parent filled-in the formula) and from the same teacher. In a case the teacher had left/changed between the different measurement points, only the grades and absence from school of students were asked (from the new teacher).

## Measurements

### Students

#### Questionnaire

A comprehensive set of standardized questionnaires is being filled in by all participants (Table 1).

**Table 1:** Outcome measures

Outcomes	Informant			Measurement
	Student	Teacher	Parent	
Mental Wellbeing				
*Primary outcomes*				
Resilience, resilience scala (RS14)	x			
Existence or absence of depressive symptoms (RBDI)	x			
Experienced psychological strenghts and difficulties (SDQ)	x	x	x	
*Secondary outcomes*				
Mindfulness (CAMM)	x			
Happiness (OECD Better life Index)	x			
Satisfaction with life (SWLS-C)	x			
Quality of life (KINDL-R)	x		x	
Positive and negative affects (PANAS)	x			
The Rumination-Reflection Questionnaire				
Stress in Children (SIC Qestionnaire)				
Compassion/self-kindness)	x			
Cognitive measuments				
NEPSY-II (Developmental Neuropsychological Assesment)				x
WISC-IV (Wechsler Intelligence Scale for Children)				x
D-KEFS (Delis-Kaplan Executive Function System)				x
Psychological flexibility (CERQ)	x			
Viivi, 5-15 questionnaire on child development			x	
Psycho-physiological responses				
SCR (Skin conduct response)				x
EKG (Electrocardiography)				x
Academic achievement/school				
Grade average in the last school report	x	x		
Grades in last school report	x	x		
Satisfaction with ow achievements	x			
Days of absence from school		x		
Bullying at school	x			
Health behavior in school-aged children, WHO HBSC				
Physical activity	x			
Sleeping/tiredness	x			
Alcohol use	x			
Smoking	x			
Screen time	x			
Motivational determinants of practice				
Outcome expectations	x			
Use of strategies to relax	x			
Self-efficacy	x			
Intention/motivation	x			
Class room social environment (CES)	x	x		
Personality inventory (TIPI)	x			
Psycho-social background factors				
Experienced major difficulties in life	x			
The quality of social relationship with peers	x			
Major changes in student's life			x	
The emotional athmospere at home	x		x	
The relationship with mother	x			
The relationship with father	x			
Socio-demopraphic background factors				
Financial situation in the family	x			
Family composition	x			
Mother tongue	x		x	
Parent education			x	
occupation			x	
Employment status			x	

Students fill in their questionnaires at school under facilitators’ or teachers’ monitoring. Parents fill in their questionnaires at home and bring/send them to school in a closed envelope. Teachers fill in their questionnaires during their working hours at school, if possible. The filled questionnaires (students, teachers, parents) are collected from schools and brought to recording company’s premises approximately 2–3 weeks after the intervention period has finished.

##### Primary outcomes

In children’s and adolescents, existence or absence of depressive symptoms was measured with the Finnish version of the Beck Depression Inventory (RBDI) [57]. The well-being was measured with the Strenghts and Difficulties Questionnaire (SDQ) [58]. The resilience was measured with Resilience Scale (RS14) [59] that has shown good internal consistency reliability among adults, Cronbach Alpha (CA) 0.87 [60]. The Finnish versions of SDQ [61], Cronbach Alpha (CA) 0.71 and RBDI [62, 63] CA 0.83, 0.87 have shown adequate psychometric properties among youth.

##### Secondary outcomes

The secondary outcomes of the present study are conceptualized as children’s and adolescents’ cognitive–emotional factors that are essential for their resilience, mental health and well-being; Mindfulnesss, Happiness, Satisfaction with Life, Quality of Life, Positive and Negative affects, and compassion/self-kindness, the rumination, and stress. Additionally cognitive functions, psychophysiological responses, academic achievements, health behavior, and motivational determinants of practice have been included in the present study (Table 1).

##### Psycho-physiological and neuropsychological measures

Both the objective neuropsychological and psychophysiological measures were collected from a subset of students: 62 students in the intervention group and 69 students in the control group (relaxation programme) were randomly selected from four 6th grade and four 8th grade classes (*N=*131). There were three measurement points: before the intervention started , directly after the intervention period, and 6 months after the intervention period. Neuropsychological tests include subtests from NEPSY-II [64], WISC-IV [65] and D-KEFS [66].

*NEPSY-II* [64] (Developmental Neuropsychological Assessment) is a series of neuropsychological tests, used in various combinations to assess neuropsychological development in children [64]. In this study we will administer the test of *Inhibition*, measuring the ability to inhibit and switch response types, which is a part of the attention and executive functioning domain category.

*WISC-IV* (Wechsler Intelligence Scale for Children) is a well-known and widely used assessment of cognitive functioning in children [65]. We administer the *Working memory* subtest, which assesses the ability to hold and manipulate new information in the short-term memory.

*D-KEFS* (Delis-Kaplan Executive Function System) is set of neuropsychological tests used to measure variety of verbal and non-verbal executive functions [66]. The subtests to be administered include the *Trailmaking test* (measuring flexibility of thinking on a visual-motor sequencing task) and the *Verbal fluency test* (measuring letter, category and category switching fluency).

##### Psycho-physiological measures

The psycho-physiological measurement will be conducted with the mobile Nexus instruments from the psychology laboratory in Helsinki University. The measurement includes skin conductance response, heart rate and electrocardiography.

Skin conductance response [67] method for measuring the electrical conductance of the skin which varies with moisture level. Sweat glands are controlled by the sympathetic nervous system, so skin conductance is used as an indication of psychological or physiological arousal. Therefore, if the sympathetic branch of the autonomic nervous system is highly aroused, sweat gland activity will also increase, which in turn increases skin conductance. In this way, skin conductance can be used as a measure of emotional and sympathetic responses. A pair of electrodes is attached to palm or fingers to measure the response over a period of time.

Electrocardiography is a transthoracic interpretation of the electrical activity of the heart over a period of time, as detected by electrodes attached to the surface of the skin and recorded by an electrocardiogram [68]. The electrical activity of the heart is sensitive to the changes of a range of bodily functions, such as effects of the autonomic nervous system, metabolism and hormonal influences (Table 1).

### Measurement procedure

Instruments are placed in a classroom, where the students can come in groups of 3. The measurement will take approximately 1 h/student. At first there will be the basal or resting measurement. After that students will be presented two stress inducing tasks. The first task is a mathematical problem (cognitive stress) and the second task is a small speech given to the researcher, research assistants and others students (social stress). Speech task is divided to three parts, so each student has the opportunity to give their speech on a novel subject, while others listen.

### Teachers

The teacher rated secondary outcome measures include experienced psychological strengths and difficulties measured by Strengths and Difficulties Teacher Form [58], and classroom social environment measured by Classroom Environment Scale [69]. In addition to these, in 6 months’ follow-up teachers were asked to assess the pedagogical and beneficial elements of the intervention and control programs both to their students, as well as their own work load and work satisfaction (Table 1).

### Parents

Parents were asked background information regarding their education, sufficiency of their salary to necessary expenses, athmosphere at home, major life changes (of their child attending the study or the whole family) and experienced psychological strengths and difficulties measured by Strengths and Difficulties Parent Form [58]. Apart from the background information, a description of all measures used in the data collection is reported in Table 1.

### Long run follow- up

In addition to 6 and 12 months follow-up, a linkage to main health, or health related, registers will be done (The Social Insurance Institution of Finland; National Institute for Health and Welfare; Statistics Finland).

### Intervention

A 9-week mindfulness intervention program .b (Stop & Breathe) [46] is designed to teens aged 11–18 years by experienced classroom teachers and mindfulness practitioners with researchers from the Oxford, Cambridge and Exeter universities. The program consists of nine 45-min group sessions and mindfulness home practices designed to improve emotional awareness, sustained attention, and attentional and emotional regulation. The program is standardized, highly recognized; and the preliminary research, though based on small intervention populations, suggests that it is effective [49].

### Active control intervention

The control group receives a standardized relaxation program called “Relax” developed in co-operation with Folkhälsan Förbundet (based on program called “Chilla”). Relax-program aims to produce relaxation skills and holistic wellbeing for the control group attendants. Every lecture is divided in two parts, relaxation exercises and group discussion about different topics, e.g., stress, relaxation, upsides and downsides of smartphones, sleep, excercising, food and attitudes. Relaxation includes progressive muscle relaxation, a breathing excercise, visualization, choose your emotion for rest of the day and short brake for regaining energy. The dose of the program is the same as in the .b intervention, i.e. nine 45 min group sessions and home practices.

### Non-treatment control intervention

The third arm, non-treatment-group will fill-in the same research questionnaires during the same time periods as the intervention and control groups (except the short questionnaire after the 5th lesson) in spring 2015 and in spring 2016 (ongoing). The non-treatment group will receive a shorter well-being course after the one year follow-up has been conducted.

### Pilot intervention study

The acceptability and feasibility of the program has been ensured in a previous controlled pilot intervention study in two schools (4 classrooms with 19–22 students each, altogether 82 participants). The study was conducted in autumn 2012 and it indicated suitability and fit of the program to the Finnish educational system, students and staff. A qualitative assessment and the quantitative calculations showed promising effects on pupils’ executive skills and well-being. Quantitative analysis showed differences between genders; among girls the greatest benefits were seen in improved self-esteem (*p* = 0.008) and stress resilience (*p* = 0.014), whereas among boys in improved self-awareness (*p* = 0.006).

### Treatment fidelity

The program is delivered by 14 educated facilitators. All facilitators were provided with a self-monitoring sheet which are used for the self-assessment of their performance (e.g. intention, attitude, ability to be mindful and conduct the lesson with empathy and kindness) as well as to guarantee that the core elements of each lesson are delivered. The facilitators also assess the student’s behaviour and ability to receive and internalize the core elements of a given .b lesson, as well as the teachers’ presence at lessons and attitudes toward the program.

Before the intervention data collection was launched, each facilitator conducted a randomly selected .b lesson that was assessed both quantitatively and qualitatively by research group members and collagues who have attended a mindfulness-based stress reduction course but who are not part of the present research group. These lessons were also videotaped, as well as the mentioned assessment discussion. This procedure was conducted to guarantee that all facilitators are conducting “the same program with the same intention”. Out of the 14 facilitators, all nine intervention group facilatators have attended a 8-week mindfulness-based stress reduction course, are educated in delivering .b school program, and practice mindfulness in their own lives. All facilitators, including active control group facilitators, except one, have received their basic education either in education or health and welfare, consisting of teachers (5), psychologists (2), health professionals (5), nutritionist (1), and a lawyer (1).

## Sample size

The sample size was estimated to detect the mean difference of 0.2 standard deviation units (effect size = 0.2) on main outcomes of risk for depression (RBDI), social/emotional/behavioural skills (SDQ) and resilience (RS14) between intervention and control groups with 80 % power and the two-tailed 5 % level of significance. The clustering of outcomes within schools was taken into account, assuming an intra-cluster (intra-school) correlation coefficient of 0.03 and assumed that on average 60 children in each school will complete the study. The required sample size was estimated to be 1090 children per group, and allowing for about 10 % drop-out rate, the study requires 1200 children per group and total of 2400 children to be recruited. On the RBDI and SDQ total difficulties score, an effect size of 0.2 corresponds to a mean decrease of 0.8 score on the RBDI scale and a mean decrease of 1.0 score on the SDQ scale, assuming the standard deviations of 4 for RBDI [62] and 5 for SDQ [61]. The effect size of 0.2 corresponds to a mean increase around 2.5 score on the resilience scale, assuming the standard deviation of 13 [70].

In addition to comparing the intervention and control groups, we were interested in comparing the intervention and non-treatment groups in order to gain even more strength into the study design. However, this was not our primary intrest. Since the previous research has shown also standard relaxation programs to have beneficial effects on well-being, we are expecting to find greater differences between intervention and non-treatment groups compared to intervention and control groups. Using the same assumptions to detect the mean difference of 0.3 standard deviation units (effect size of 0.3) between intervention and non-treatment group, the required sample size was estimated to be 486 children per group, and allowing for about 10 % drop-out rate, the study requires 540 children in the non-treatment group.

### Material management

Questionnaires are stored in a locked-up room and closet at the Folkhälsan Research Center. Data is transferred into a digital format and analyzed anonymously using an identification number given for each participant, not allowing for personal identification, and is managed by designated, trained personnel. Only selected members of the research group have access to the data.

## Analysis plan

Data will be analyzed on an intention to treat basis including all randomized classes in the groups to which they were randomly assigned. Descriptive statistics (mean, median or percentages as appropriate) will be used to summarize the baseline characteristics and outcomes in each group.

Statistical analysis will be done with multilevel (hierarcial) models to account for the clustering within schools. Continuous outcomes will be analysed with linear mixed effects models and categorical outcomes with generalized linear mixed effects models. Maximum likelihood estimation will be used to get unbiased and efficient parameter estimates for data with missing values in the follow-up measurements.

The effectiveness of the mindfulness intervention on primary and secondary outcomes will be first analyzed using unadjusted analyses and then adjusted for age, sex and baseline values of the outcomes. The modifying effect of factors (i.e. sex, childen’s age, health status, circumstances at home, social relationships, hobbies, school achievement) on the effectiveness of mindfulness will be analysed using tests of interactions. Interaction analyses are exploratory in nature. The differences in the continuous outcomes between groups will be presented using mean differences with 95 % confidence intervals. Results are expressed using odd ratios with 95 % confidence intervals for categorical outcomes. Two-sided statistical tests with a 5 % level of significance will be used.

## Discussion

This paper describes the rationale and design of a cluster randomized controlled trial of a mindfulness intervention program among children and adolescents compared to an active control group receiving standard relaxation program, and a non-treatment group. The trial presented in this protocol aims to expand our knowledge on the effectiveness of mindfulness on a variety of behavioral, emotional, cognitive, and psychophysiological outcomes, compared to an alternative treatment and no treatment at all.

By testing the effectiveness of two alternative strategies for promoting human resilience and well-being, the present research will eventually offer new insight into the comparative usefulness of mindfulness interventions. We also focus on the unresolved questions of the mindfulness research by using a systematic and sound design to avoid methodological shortcomings.

To our knowledge, the present study is among the first ones to conduct systematic, methodologically rigorous comparative randomized research among school-aged children, on the effects of mindfulness on mental well-being.

## Abbreviations

No abbreviations used.
